# Medicine and the Public

**Published:** 1905-10-14

**Authors:** 


### The Hospital.

A Journal of

The Medical Sciences and Hospital Administration.

Vol. XXXIX.—No. 994. SATURDAY, OCTOBER 14, 1905.

### MEDICINE AND THE PUBLIC.

The members of the medical profession have formed an integral part of society in the United Kingdom for so long a time that, in legal phraseology, the memory of man runneth not to the contrary. Their numbers have always been considerable; their presence and their ministrations have been in frequent request in the great majority of families; they have presented, like other sections of the community, all varieties of character, of disposition, and of capacity; and yet, notwithstanding the abundant experience of them thus afforded, they still offer an insoluble riddle to large numbers of well-meaning people. They are a stumbling-block to the novelist, a mystery to the most omniscient newspaper man, and a source of continual trouble and perplexity to the gossip. Not long ago we heard an account of them in a railway carriage. The narrator was an old gentleman over whose whole person half-pay officer was writ large, and who manifestly belonged to that school whose firmest conviction is that the Service is going to the dogs. An acquaintance entered the carriage, and a somewhat loud conversation commenced. The government, the directors of the railway on which we were travelling, the weather, and the taxes, were successively taken in hand and dismissed with a few words of earnest condemnation, and then came the turn of the doctors. The old gentleman was in distress at the recent death of a friend, who, he said, had been to consult "one of those London fellows," had paid him a large fee, had received an assurance of being in perfect health, so that he might "go home and enjoy himself," and had died suddenly two days afterwards. "That is the way," continued the old gentleman, "with all these doctors. They don't know anything about you, how should they? but they give you a dig in the ribs, tell you to put out your tongue, ask two or three questions, say there is nothing the matter, and hold out their hand for five guineas; and they do that forty times in a morning. And then you go home and die in two days."

A picture which may, perhaps, be regarded as a pendant to the above was supplied to a morning newspaper, last week, by that ingenious person, Mr. H. G. Wells, whose conceptions of a futurity under the reign of what he fancies to be science have probably been glanced at by some of our readers. Mr. Wells looks forward, as the late Sir Benjamin Ward Richardson did before him, to a time when all doctors will be members of one great organisation for the public health, with all or most of their incomes guaranteed to them; and in the meanwhile he provides us with sketches of medical education and of medical practice so grossly caricatured that it is difficult to conceive how any man living in the world with his eyes open can have sufficient assurance to offer them to readers. We are told that "for the great majority of the medical profession there is no living to be got except *as a salary for hospital practice* or by earning fees in receiving or attending upon private cases. His (the doctor's) four or five years of crowded study are supposed (we are not told by whom) to give him *a complete and final knowledge of the treatment of every sort of disease*, and he goes on year after year, often without co-operation, working mechanically in the common incidents of practice, births, cases of measles and whooping cough and so forth, and blundering more or less in whatever else turns up. There are no public specialists to whom he can conveniently refer the difficulties he constantly encounters, there are no properly organised information bureaux for him, and *no means whatever* of keeping him informed upon progress and discovery in medical science." In Mr. Wells's Utopia, whenever it comes into existence, for every medical man who is engaged in practice there is to be another who is mainly occupied in or about research, and then all manner of wonderful results are to be obtained.

It is melancholy to think that such drivel as this can be supplied by a newspaper proprietor to his subscribers as matter which they may fairly be asked to read and to consider. We can excuse Mr. Wells for having no apparent knowledge of the existence of the numerous scientific societies dealing with different departments of medicine, and having for their chief function the work of keeping the members informed upon "progress and discovery in medical science." We can excuse him for not knowing that the innumerable hospitals provide "public specialists" for the poor, probably far in excess of the actual requirements of the case. We can excuse him for his apparent belief that medical men are paid for their hospital work, even to the extent of its furnishing them with a means of living; although any printer's devil could have told him that such work is, with a very few insignificant exceptions, wholly unpaid. But it is absolutely inexcusable for a professed teacher of mankind to think of "research" as if it were something which could be conducted to trustworthy conclusions, by any considerable number of people. The intellectual capacities required for the fruitful conduct of research are among the rarest of the gifts of nature to mankind; and the few who possess them stand out from among their contemporaries, in every generation, as men apart. Newton, Young, Faraday, Pasteur are the types which the idea of research recalls to the scientific mind; and the suggestion that men of corresponding type are ever to constitute one-half of the total number of the medical profession is either the most extravagant compliment which that profession ever received, or else the most convincing possible proof of the futility of Mr. Wells's speculations.

